# Emergency Carotid Thrombo-Endarterectomy after Failed Endovascular Recanalization for Acute Complete Carotid Occlusion: A Case Report

**DOI:** 10.3390/brainsci14090882

**Published:** 2024-08-30

**Authors:** Abdullah Keles, Zeynep Arzum Uyaniker, Beverly Aagaard-Kienitz, Mustafa K. Baskaya

**Affiliations:** Department of Neurological Surgery, School of Medicine and Public Health, University of Wisconsin-Madison, Madison, WI 53792, USA; abdullah.keles@wisc.edu (A.K.); arzumuyaniker@gmail.com (Z.A.U.); baagaard-kienitz@uwhealth.org (B.A.-K.)

**Keywords:** carotid artery stenting, carotid endarterectomy, carotid occlusion, emergency, endovascular recanalization, stroke, thrombo-endarterectomy

## Abstract

Rapid identification of the type and origin of a stroke is crucial for prompt and appropriate treatment, which can significantly influences patient outcomes. We report a multidisciplinary management case involving a 76-year-old man who presented with left-sided weakness and mild dysarthria. Imaging revealed a completely occluded right internal carotid artery. Despite multiple endovascular recanalization attempts, adequate flow could not be achieved, leading to the decision to perform an open thrombo-endarterectomy. The patient underwent carotid endarterectomy with microsurgical techniques under general anesthesia. The atheroma plaque and central thrombus were removed, which reestablished flow. Continuous intraoperative neuromonitoring was utilized to ensure patient safety. The patient woke up without new deficits and was discharged for rehabilitation. Follow-up imaging confirmed arterial patency, and the patient eventually made an excellent recovery, including being independent over one and a half years. Emergent recanalization with carotid endarterectomy following a failed endovascular recanalization is both safe and feasible, emphasizing the need for collaboration between different treatment providers to ensure optimal patient outcomes. Our report highlights the importance of a multidisciplinary approach and the advantages of a hybrid operating room in the treatment of acute complete carotid artery occlusion.

## 1. Introduction

The identification of stroke type and origin are crucial for providing prompt and appropriate treatment, the most important determinants of a better outcome. The majority of strokes are of ischemic origin, a severe clinical condition that leads to significant neurological impairment and a high mortality rate. Notably, more than 20% of ischemic strokes are attributed to carotid artery occlusive disease [1,2].

Hunt et al. were the first to describe carotid artery syndrome in ischemic strokes, characterized by a weak carotid pulse, contralateral paresis, and ipsilateral monocular blindness. They emphasized the importance of routine examinations of the carotid vessels in the neck on the side contralateral to the hemiplegia [3,4,5]. Efficient carotid arteriography, introduced by Moniz, has brought attention to this syndrome and increased the frequency of diagnosing internal carotid occlusion [5,6].

Beginning in the 1950s, carotid endarterectomy (CEA) became widely adopted and has been demonstrated to be effective in restoring blood flow and preventing recurrent strokes in patients with complete stenosis.

Emergency CEA may be necessary to restore neurological function, address the risk of further neurological deficits, or both, due to acute complications, acute complete occlusion, or failed endovascular carotid artery stenting (CAS).

In this report, we emphasize the significance of a multidisciplinary approach in treating acute carotid artery occlusion. Our case also highlights the advantages of a hybrid operating room, which enabled us to seamlessly transition from our endovascular colleagues to perform an emergency CEA in the same session without any treatment delay.

## 2. Results

### 2.1. Case

A 76-year-old man was admitted to an outside institution with left-sided weakness. The patient had a recent diagnosis of atrial fibrillation. He was otherwise healthy, with no past medical or surgical history. On the initial neurological examination, he was found to have mild dysarthria, a left facial droop, left pronator drift, left upper limb strength of 3/5, and left lower limb strength of 4/5. The rest of his neurological examination was normal.

**Figure 1:**
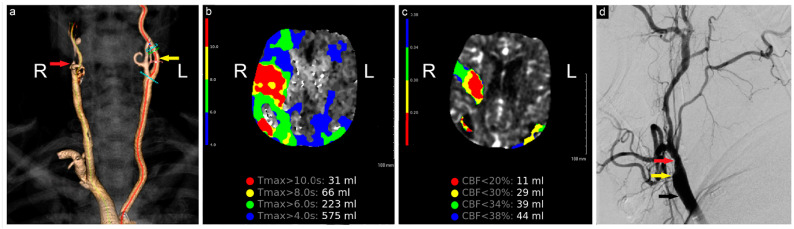
Preoperative neuroimaging. (**a**) CTA shows complete occlusion of the right ICA; (**b**) computed tomography perfusion study shows a large region of T_max_ > 6 s (time to maximum of the tissue residue function) measuring 223 mL, primarily involving the right middle cerebral artery territory, (**c**) with a smaller region of CBF< 30% measuring 29 mL in the same territory. The mismatch volume and ratio were 194 mL and 7.7, respectively, with a hypoperfusion index of 0.1; (**d**) angiogram shows complete occlusion of the right ICA (yellow arrow), patent ECA (red arrow), and CCA (black arrow). R: Right, L: Left, CBF: Cerebral Blood Flow.

The initial computed tomography angiography (CTA) at an outside hospital showed a completely occluded right internal carotid artery (ICA). The patient was loaded with aspirin and transferred to our institution for further evaluation and treatment. On arrival, additional neuroimaging studies were conducted (Figure 1). Endovascular and open cerebrovascular services were then consulted for a possible thrombectomy and recanalization intervention.

All treatment options were discussed with the patient and the family, and the decision was made to proceed with endovascular recanalization, including stenting with or without angioplasty, and thrombectomy. The patient initially underwent an attempted endovascular recanalization and thrombectomy by the endovascular team in the hybrid operating room five hours after the initial symptoms occurred. Our endovascular team routinely and successfully re-opens completely occluded cervical ICA in acute stroke treatment, using a triaxial system and crossing the occluded lumen first with a microwire. However, in this case, multiple attempts to cross the occlusion with the microwire were unsuccessful due to the stiffness and large size of the atheroma plaque, which made recanalization particularly challenging. Given the patient’s presentation with acute ischemic stroke, the inability to cross the carotid occlusion endovascularly, and the immediate availability of a cerebrovascular surgeon, the decision was made to proceed with open endarterectomy, which was promptly performed in the same session.

#### 2.1.1. Procedure

**Figure 2:**
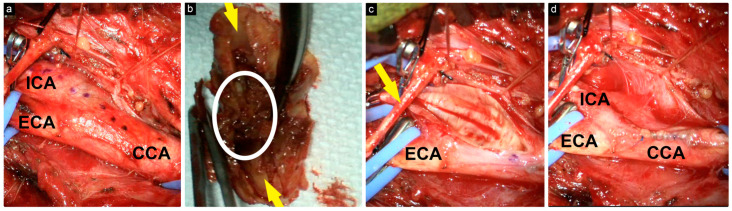
Intraoperative view of the surgical field. (**a**) CCA, ECA, and ICA were carefully dissected, and the incision length was marked with a dotted line; (**b**) excised atheroma plaque with central thrombus (encircled) and the lumen (yellow arrows); (**c**) exposure after atheroma plaque removal; the hypoglossal nerve (yellow arrow); (**d**) exposure after the closure of the arteries with 6-0 Prolene.

Following the induction of general anesthesia, the patient was positioned in the standard supine position. The incision was marked, and the surgical field was then draped. The skin and platysma were incised, and the fascia was entered. The common carotid artery (CCA), external carotid artery (ECA), superior thyroid artery, and ICA were then visualized and mobilized. The hypoglossal nerve was identified and preserved (Figure 2).

After the exposure of all arteries was complete, we checked for flow using a micro-Doppler on the distal ICA to determine whether the artery distal to the occlusion point was patent. Since we had good Doppler signals at the ICA distal to the occlusion, we decided to proceed with the endarterectomy and recanalization.

The CCA, ECA, ICA, and superior thyroid artery were then clamped. Before cross-clamping, we administered 7000 units of intravenous heparin.

A longitudinal incision was made just inferior to the carotid bifurcation, and the atheroma plaque and central thrombus were exposed. We carefully identified the plane between the plaque and the outer wall and dissected the plaque using dissectors. It was then cut from the CCA and the ECA, and finally removed from the ICA until normal intima was reached.

Following the removal of the plaque, the lumen of the artery was thoroughly irrigated with a heparin solution. We then obtained good backflow through the ICA by temporarily opening the clip.

The arteriotomy was closed with 6-0 Prolene in a running fashion. Just before tying both the proximal and distal sutures, backflow was allowed to flush out intravascular air and debris by temporarily unclamping the ICA. We confirmed good flow in all vessels with micro-Doppler. The platysma, subcutaneous layers, and skin were then closed in layers.

Throughout the procedure, we utilized continuous somatosensory evoked potentials (SSEPs) and motor-evoked potentials (MEPs), electroencephalography (EEG) neuromonitoring, as well as micro-Doppler ultrasonography. We also maintained an average systolic arterial pressure of 160 mm Hg.

In cases where we observe any changes in the EEG, MEPs, or SSEPs, we raise the systolic arterial pressure to 200 mm Hg. If the changes are not reversed, we consider inserting the shunt, which is always kept available. Postoperative neurological complications can be avoided to a great extent with close and continuous intraoperative neuromonitoring and management.

#### 2.1.2. Early Postoperative Course and Follow-Up

The patient woke up without any new deficits, and the postoperative course was uneventful. He was placed on a daily regimen of aspirin (325 mg) and clopidogrel (75 mg). He progressed well throughout his hospitalization and was discharged to acute rehabilitation for further physical and occupational therapy.

**Figure 3:**
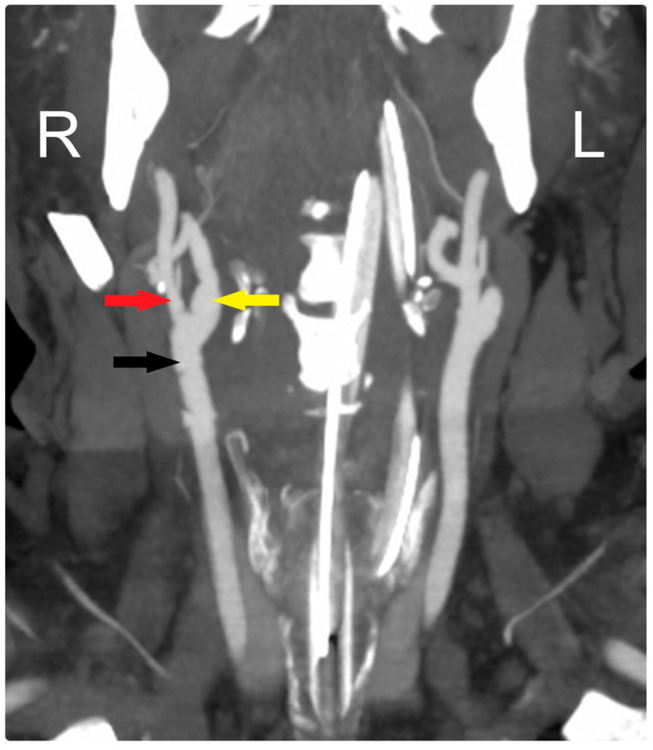
Early postoperative CTA shows patency of all arteries: CCA (black arrow), ECA (red arrow), and ICA (yellow arrow). R: Right, L: Left.

At his early postoperative period, a CTA scan of the head confirmed the patency of the CCA and ICA (Figure 3). Over a one-and-a-half-year follow-up period, the patient became neurologically intact and was functioning independently, with the arteries remaining patent throughout this time.

## 3. Discussion

Despite medical treatment, proximal large vessel occlusion in the anterior circulation causes up to 80% mortality or severe neurological impairment within three months after a stroke, making it the subtype with the worst prognosis [7,8,9,10].

The current management of carotid artery occlusion includes medical treatment, CEA, and CAS. Recent evidence, such as the study by Loufopoulos et al., continues to support CEA as superior to CAS in reducing the incidence of new cerebral ischemic lesions post-procedure, reaffirming the long-standing role of CEA as the gold standard in appropriate cases [11].

Medical therapy might be considered for patients who decline endarterectomy or stenting, have disease not suitable for these interventions, or need treatment during the period between diagnosis and intervention [12]. However, large-scale randomized clinical trials have shown a slight advantage for open surgery compared to the best medical therapy alone in patients with significant asymptomatic carotid stenosis [13,14].

Historically, the surgical management of carotid occlusion included arterectomy, cervical sympathectomy, and thrombo-endarterectomy [3,5]. Since the 1950s, CEA has been widely implemented to reestablish blood flow and prevent recurrent strokes in patients with complete carotid artery stenosis. The indications for CEA in both symptomatic and asymptomatic carotid artery occlusion were clearly established through large, prospective, multicenter randomized clinical trials [14,15,16]. Sastry et al. confirm the superior safety profile of CEA compared to CAS in reducing periprocedural stroke risk, especially in older patients, adding new dimensions to the patient selection criteria [17].

In the 1990s, CAS appeared as a potential less invasive treatment option compared to CEA and was initially utilized for patients who were ineligible for CEA [18,19]. Since then, it has gained wide usage in practice, with overall utilization increasing from 2.8% to 12.6% of all carotid stenosis revascularization procedures from 1998 to 2008 [20]. However, emerging data from Gao et al. suggest that, while both CEA and CAS are effective for treating asymptomatic carotid stenosis, CEA remains the more effective treatment in terms of reducing stroke risk, particularly in patients with significant stenosis [21].

### 3.1. Randomized Trials Comparing CEA and CAS

Multiple randomized trials have yielded inconsistent results regarding the overall safety of CAS versus CEA. While some studies indicate that CAS is not inferior to CEA, none have shown that CAS is superior in terms of perioperative safety [22,23,24,25,26,27,28,29,30,31,32].

According to Dumont et al. and Singh et al., complications such as stroke, myocardial infarction, and space-occupying hemorrhage occur more frequently with CAS, which also has a higher perioperative incidence of stroke and death, compared to CEA [20,33]. However, Bonati et al. found that CAS is associated with reduced risks of myocardial infarction, cranial nerve palsy, and access site hematoma [30]. A meta-analysis of randomized controlled trials conducted by Li et al. found that, despite being associated with a higher risk of myocardial infarction, CEA was superior for both short-term and long-term outcomes [32]. Additionally, long-term follow-ups revealed significantly higher restenosis rates for CAS compared to CEA [34,35]. Conversely, different studies found similar long-term functional outcomes for patients undergoing either CEA or CAS [30,31,36].

Recent work by Loufopoulos et al. further strengthens the argument for CEA, showing that across 25 studies, the rate of new cerebral ischemic lesions was significantly lower after CEA compared to CAS at all time points post-procedure. These findings suggest that CEA should remain the first-line treatment, especially in patients with higher stroke risk profiles, despite CAS being a less invasive alternative [11].

Despite CEA being the gold standard for decades, both techniques are now recognized as effective treatments in selected cases and are commonly used in current practice. Since CAS is less invasive and has a shorter procedure time, it is more beneficial for selected cases such as carotid dissection, ICA stenosis post-radiotherapy, and patients with comorbidities. The study by Sastry et al. highlights that CAS may be more suitable for younger patients and those at lower stroke risk, given its association with fewer cardiac complications and reoperation rates. However, the same study also notes that CAS shows an increasing stroke risk over time, particularly in older populations, reinforcing the importance of careful patient selection [17]. The selection of treatment should be tailored to individual patient factors, procedural risks, and the availability of the medical team.

### 3.2. Emergency CEA

Early experimental studies on cerebral ischemia revealed that it is a progressive condition that can be reversed if intervention occurs in the initial stages [37,38,39]. Early revascularization is a well-known predictor of positive outcomes in acute ischemic stroke, and every 30-min delay can decrease the chances of a favorable outcome by 10% [40,41,42]. According to Ojemann et al., the ideal treatment window for treating an acute complete occlusion of the carotid artery due to thrombus is within 12 to 24 h following the presumed time of complete occlusion [43].

Although emergency CEA has been performed by different groups for a long time, the criteria can vary [44,45,46]. Emergency surgical intervention may be necessary to restore neurological function, address the risk of further neurological deficits, or both, due to acute complications, acute complete occlusion, or failed CAS. Savardekar et al. reinforce the benefits of urgent CEA in symptomatic carotid stenosis within 48 h of a TIA or stroke, although the precise timing remains a subject of debate. The paradigm is shifting towards early CEA, especially in cases of TIA as the index event [47].

As demonstrated in our case, neurosurgeons in institutions offering both CAS and CEA treatment options should be adaptable and work collaboratively with their colleagues to determine the best treatment approach for each specific case.

Depending on the chosen treatment method for carotid occlusion, prompt intervention in case of complications or treatment failure is crucial. This case highlights the critical importance of a multidisciplinary approach and a hybrid operating room, where both endovascular and open vascular procedures can be performed, allowing for real-time strategic adjustments to ensure optimal patient outcomes.

## 4. Conclusions

As demonstrated in our case report, early recanalization with CEA following a failed CAS is both safe and feasible and should be considered for treating acute complications, occlusions, or failed CAS. Additionally, our reports emphasize the significance of collaboration between providers of both treatment modalities and the use of a hybrid OR, which enabled us to perform CAE immediately after a failed CAS in the same setting.

## Data Availability

The original contributions presented in the study are included in the article/supplementary material, further inquiries can be directed to the corresponding author.

## Supplementary Materials

The following supporting information can be downloaded at: https://www.mdpi.com/article/10.3390/brainsci14090882/s1, Video S1: Emergency carotid thrombo-endarterectomy after failed endovascular recanalization for acute complete carotid occlusion: a surgical video illustration.
